# A geometric relationship of *F*_2_, *F*_3_ and *F*_4_-statistics with principal component analysis

**DOI:** 10.1098/rstb.2020.0413

**Published:** 2022-06-06

**Authors:** Benjamin M. Peter

**Affiliations:** Max-Planck-Institute for Evolutionary Anthropology, Leipzig 04103, Germany

**Keywords:** population structure, principal component analysis, *F*-statistics

## Abstract

Principal component analysis (PCA) and *F*-statistics *sensu* Patterson are two of the most widely used population genetic tools to study human genetic variation. Here, I derive explicit connections between the two approaches and show that these two methods are closely related. *F*-statistics have a simple geometrical interpretation in the context of PCA, and orthogonal projections are a key concept to establish this link. I show that for any pair of populations, any population that is admixed as determined by an *F*_3_-statistic will lie inside a circle on a PCA plot. Furthermore, the *F*_4_-statistic is closely related to an angle measurement, and will be zero if the differences between pairs of populations intersect at a right angle in PCA space. I illustrate my results on two examples, one of Western Eurasian, and one of global human diversity. In both examples, I find that the first few PCs are sufficient to approximate most *F*-statistics, and that PCA plots are effective at predicting *F*-statistics. Thus, while *F*-statistics are commonly understood in terms of discrete populations, the geometric perspective illustrates that they can be viewed in a framework of populations that vary in a more continuous manner.

This article is part of the theme issue ‘Celebrating 50 years since Lewontin's apportionment of human diversity’.

## 1. Introduction

As in most species, the genetic diversity of human populations has been influenced by our history and environment over the last several hundred thousand years [e.g 1–5]. In turn, an important goal of population genetics is to use observed patterns of variation to investigate and reconstruct the demographic and evolutionary history of our species [6,7].

The complicated genetic structure observed in present-day human populations [8,9] is caused by the interplay of demographic and evolutionary processes with both discrete and continuous components [10–14]. In particular, populations are expected to differentiate if they are isolated from each other [15,16]. In humans, this may be caused because continental-scale geographic distances limit migration, causing a pattern known as isolation-by-distance [17,18]. However, these patterns are usually not uniform, but shaped by geography, particularly barriers to migration such as mountain ranges, oceans or deserts [1,19]. In addition, major historical population movements such as the out-of-Africa [20], Austronesian [21] or Bantu expansions [22] lead to more gradual patterns of genetic diversity over space [23]. Local migration between neighbouring populations will reduce differentiation, and long-distance migrations [24], and secondary contact between diverged populations such as Neanderthals and modern humans [25] may lead to locally increased diversity [26].

Particularly for large and heterogeneous datasets, disentangling all these processes is challenging, and we cannot expect to devise a single model catching both broad strokes and minute details of human history. A commonly used analysis paradigm is thus to combine tools based on different sets of assumptions, each emphasizing particular aspects of the data.

A typical analysis starts with data-driven, exploratory methods that summarize data making minimal assumptions [e.g. 6]. Examples are population trees [16,27,28], principal component analysis (PCA [1,29,30]) structure-like models [31,32] or multidimensional scaling (MDS [33]). These methods are limited in their ability to estimate biologically meaningful parameters, but provide useful summaries and visualizations. Typically, these analyses are then complemented with methods based on explicit demographic models, which are used to estimate parameters or test hypotheses [34–36].

**Figure 1.**
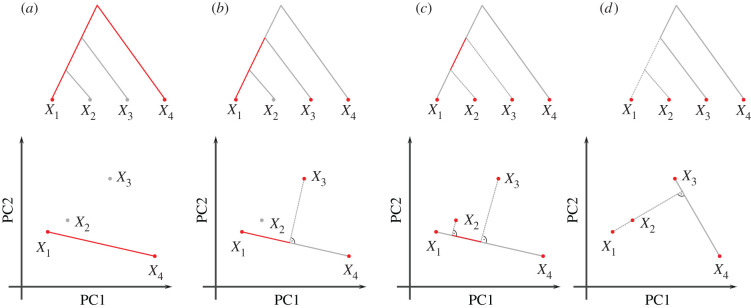
Representation of *F*-statistics on trees and two-dimensional PCA plots. The schematics show four populations and their representation using an (arbitrarily rooted) tree (top row) or a two-dimensional PCA plots (bottom row). (*a*) *F*_2_ represents the squared Euclidean distance between two tree leafs, and in PC space. (*b*) *F*_3_(*X*_1_; *X*_3_, *X*_4_) corresponds to the external branch from *X*_1_ to the internal node joining the populations, and is proportional to the orthogonal projection of *X*_1_ − *X*_3_ onto *X*_1_ − *X*_4_. (*c*) *F*_4_(*X*_1_, *X*_4_; *X*_2_, *X*_3_) corresponds to the internal branch in the tree, or to the orthogonal projection of *X*_2_ − *X*_3_ onto *X*_1_ − *X*4. (*d*) *F*_4_(*X*_1_, *X*_2_; *X*_3_, *X*_4_). The two paths from *X*_1_ to *X*_2_ and *X*_3_ and *X*_4_ are non-overlapping in the tree, which corresponds to orthogonal vectors in PCA space. (Online version in colour.)

When the number of populations exceeds a few dozen, even codifying reasonable population models can be prohibitively difficult. One approach is to pick a small set of ‘representative’ samples, and restrict modelling to this subset [e.g. 37,38]. However, this has the drawback that a large proportion of the available data remains unused. An increasingly popular alternative approach, particularly in the analysis of human ancient DNA, is therefore to focus on the relationship between two, three or four populations, commonly using *F*-statistics *sensu* Patterson [39–41]. Formal definition will be given in the Theory section; but an informal motivation starts with the null model that populations are related as a tree, in which each *F*-statistic measures the length of a particular set of branches (figure 1; [41,42]).

**Figure 2.**
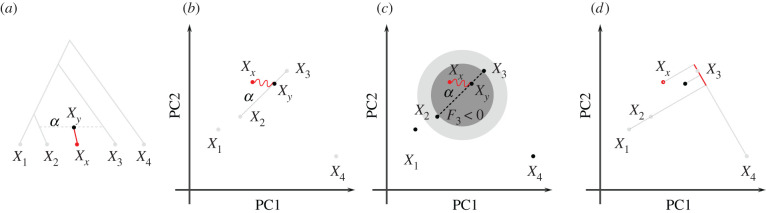
Admixture representation on two-dimensional-PCA plot. The schematics show four populations and their representation using an admixture graph (*a*) or a two-dimensional-PCA plot. (*a*) Admixture graph, with population *X*_*y*_ originating as an admixture of *X*_2_ (with proportion 1 − *α*) and *X*_3_ (proportion *α*). Subsequent drift (highlighted branch) will change allele frequency to sampled admixture population *X*_*x*_. (*b*) PCA representation of the scenario in (*a*). *X*_*y*_ originates on the segment connecting *X*_2_ and *X*_3_, and subsequent drift may move it in a random direction. (*c*) Negative region (light grey) for *F*_3_(*X*_*x*_; *X*_2_, *X*_3_) and for ${\hat{F}}_{3}^{\left(2\right)}(X_{x};X_{2},X_{3})$ based on two dimensions (dark grey). (*d*) *F*_4_(*X*_1_, *X*_*x*_; *X*_3_, *X*_4_) will no longer be zero (compare to figure 1*d*). (Online version in colour.)

In most applications, *F*-statistics are estimated from data, and then used as tests of treeness. In particular, under the assumption of a tree, *F*_3_ is restricted to be non-negative, and many *F*_4_-statistics will be zero [40,42], and data that violates these constraints is incompatible with a tree-like relationship between populations. The canonical alternative model is an admixture graph (or phylogenetic network) [40,43], which is a tree which allows for additional edges reflecting gene flow (figure 2*a*). However, admixture graphs are not the only plausible alternative model, and expected *F*-statistics can be calculated for a wide range of population genetic demographic models [41].

### (a) *F*-statistics and principal component analysis

The practical issue addressed in this study is how *F*-statistics can be compared with PCA, one of the most widely used data-driven modelling techniques. One way PCA can be motivated is as generating a low-dimensional representation of the data, with each dimension (called a principal component, PC) retaining a maximum of the variance present in the data. To understand population structure, the use of PCA has been pioneered by Cavalli-Sforza *et al.* [44], who used allele-frequency data at a population level to visualize genetic diversity [1]. Currently, PCA is most commonly performed on individual-level genotype data [e.g. 30,45], making use of the hundreds of thousands of loci available in most genome-scale datasets. The PCA decomposition has been studied for a number of explicit population genetic models including trees [16], spatially continuous structure [46], the coalescent [47] and discrete population models [48]. Here, in order to link PCA to *F*-statistics, I interpret both of them geometrically in *allele frequency space*, i.e. as functions of a high-dimensional Euclidean space. For *F*-statistics, this interpretation was recently developed by Oteo-Garcia & Oteo [49], and for PCA it follows naturally from the interpretation of approximating a high-dimensional space with a low-dimensional one.

In the next section, I will formally derive the connection between *F*-statistics and PCA, and show how *F*-statistics can be interpreted geometrically, with a particular emphasis on two-dimensional-PCA plots. In the Results section, I will then discuss how some of the most common applications of *F*-statistics manifest themselves on a PCA, and illustrate them on two example datasets, before ending with a discussion.

## 2. Theory

In this section, I will introduce the mathematics and notations for *F*-statistics and PCA. A comprehensive treatise on PCA is given by e.g. Jolliffe [50], a useful technical primer is Pachter [51], and a helpful guide to interpretation is Cavalli-Sforza *et al.* [1]. Readers unfamiliar with *F*-statistics may find [40,41] or [49] helpful.

### (a) Formal definition of *F*-statistics

Let us assume we have a set of populations for which we have single-nucleotide polymorphism (SNP) allele frequency data from *S* biallelic loci, and for simplicity, I will assume that there is no missing data. Let *x*_*il*_ denote the frequency of an arbitrary allele at the *l*th SNP in the *i*th population; and let *X*_*i*_ = (*x*_*i*1_, *x*_*i*2_, …*x*_*iS*_) be a vector collecting all allele frequencies for population *i*. As *X*_*i*_ will be the only data summary considered here for population *i*, I make no distinction between the population and the allele frequency vector used to represent it.

The three *F*-statistics are defined as

2.1a $$F_{2}(X_{1},X_{2})=\frac{1}{S}\sum_{l=1}^{S}{\left(x_{1l}-x_{2l}\right)}^{2}$$

2.1b $$F_{3}(X_{1};X_{2},X_{3})=\frac{1}{S}\sum_{l=1}^{S}(x_{1l}-x_{2l})(x_{1l}-x_{3l})$$

2.1c $$\mathrm{and}\;F_{4}(X_{1},X_{2};X_{3},X_{4})=\frac{1}{S}\sum_{l=1}^{S}(x_{1l}-x_{2l})(x_{3l}-x_{4l}).\;$$

The normalization by the number of SNPs *S* is assumed to be the same for all calculations and is thus omitted subsequently. Both *F*_3_ and *F*_4_ can be written as sums of *F*_2_-statistics

2.2a $$\begin{array}{r} 2F_{3}(X_{1};X_{2},X_{3})=F_{2}(X_{1},X_{2})+F_{2}(X_{1},X_{3})-F_{2}(X_{2},X_{3}) \end{array}$$

and

2.2b $$2F_{4}(X_{1},X_{2};X_{3},X_{4})=F_{2}(X_{1},X_{3})+F_{2}(X_{2},X_{4})-F_{2}(X_{1},X_{4})-F_{2}(X_{2},X_{3}).$$

Commonly, a distinction is made between statistics estimated from data (denoted with lowercase-*f*), and theoretical quantities (defined in equation (2.1)). I do not make this distinction, but will explicitly mention when I analyse statistics calculated from data.

*F*-statistics have been primarily motivated in the context of trees and admixture graphs [40]. In a tree, the squared Euclidean distance *F*_2_(*X*_1_, *X*_2_) measures the length of the path between populations *X*_1_ and *X*_2_ (figure 1*a*); *F*_3_ represents the length of an external branch (figure 1*b*) and *F*_4_ the length of an internal branch, respectively (figure 1*c*). The length of each branch can be thought of in units of genetic drift, and is non-negative [40]. Crucially, this means that *F*_4_ will be zero for pairs of populations from non-overlapping clades, which means that the tree lacks the branch corresponding to this statistic (as in figure 1*d*).

Thinking of *F*-statistics as branch lengths is useful for a number of applications, including building multi-population models [40,52], estimating admixture proportions [38,53] and finding the population most closely related to an unknown sample (‘Outgroup’-*F*_3_-statistic).

Most commonly however, *F*_3_ and *F*_4_ are used as tests of treeness [40]: negative *F*_3_ values correspond to a branch with negative genetic drift, which is not allowed under the null assumption of a tree-like population relationship. Similarly if four populations are related as a tree, then at least one of the *F*_4_-statistics between the populations will be zero [40,54].

The most widely considered alternative model is an admixture graph [40]; an example is given in figure 2*a*. Here, the (typically unobserved) population *X*_*y*_ is generated by a mixture of individuals from the ancestors of *X*_2_ and *X*_3_. Over time, genetic drift will change *X*_*y*_ to *X*_*x*_, which is the admixed population we observe. In this case, all *F*_4_-statistics involving *X*_*y*_ and both admixture sources will be non-zero, and, in some cases, *F*_3_(*X*_*y*_; *X*_2_, *X*_3_) will be negative (exact conditions can be found in [41]).

### (b) Geometric interpretation of *F*-statistics

An implicit assumption in the development of *F*-statistics in the context of admixture graphs has been that population lineages are mostly discrete, and that gene flow is rare. Recently, Oteo-García & Oteo [49] showed that this is not, in fact, necessary. Specifically, they interpret the populations *X*_*i*_ as points or vectors in the *S*-dimensional *allele frequency space* $R^{S}$. In this case, the *F*-statistics can be thought of as inner (or dot) products, and they showed that all properties and tests related to treeness can be derived in this larger space. In this framework, the *F*-statistics can be written as

2.3a $$F_{2}(X_{1},X_{2})=\frac{1}{S}\sum_{l=1}^{S}{\left(x_{1l}-x_{2l}\right)}^{2}=\frac{1}{S}\langle X_{1}-X_{2},X_{1}-X_{2}\rangle =\frac{1}{S}{\left\|X_{1}-X_{2}\right\|}^{2}$$

2.3b $$F_{3}(X_{1};X_{2},X_{3})=\frac{1}{S}\sum_{l=1}^{S}(x_{1l}-x_{2l})(x_{1l}-x_{3l})=\frac{1}{S}\langle X_{1}-X_{2},X_{1}-X_{3}\rangle$$

2.3c $$\mathrm{and}\;F_{4}(X_{1},X_{2};X_{3},X_{4})=\frac{1}{S}\sum_{l=1}^{S}(x_{1l}-x_{2l})(x_{3l}-x_{4l})=\frac{1}{S}\langle X_{1}-X_{2},X_{3}-X_{4}\rangle ,$$

where ‖⋅‖ denotes the Euclidean norm and 〈 · , · 〉 denotes the dot product. Some elementary properties of the dot product between vectors *a*, *b*, *c* that I will use later are

2.4a $$\langle a,b\rangle =\sum_{i}a_{i}b_{i}$$

2.4b ⟨a,b⟩=‖a‖‖b‖cos⁡(ϕ)

2.4c $$\langle a,a\rangle ={\left\|a\right\|}^{2}$$

2.4d and⟨a+c,b⟩=⟨a,b⟩+⟨b,c⟩,

where *ϕ* is the angle between *a* and *b*. The inner product is closely related to the vector projection

2.5 $${\;proj}_{b}a=\frac{\left\langle a,b\right\rangle }{{\left\|b\right\|}^{2}}b,$$

which is a vector colinear to *b* whose length measures how much vector *a* points in the direction of *b*. Thinking of *F*-statistics as projections also holds on trees: in e.g. a *F*_4_(*X*_1_, *X*_4_; *X*_2_, *X*_3_)-statistic (figure 1*c*), the internal branch is precisely the intersection of the paths from *X*_1_ to *X*_4_ and from *X*_2_ and *X*_3_. On trees, all disjoint paths are independent (i.e. orthogonal) from each other, and thus the external branches vanish under the projection.

One issue with the geometric approach of Oteo-García & Oteo [49] is that each SNP (commonly in the millions) adds a dimension, but high-dimensional spaces are hard to visualize, interpret and analyse. Fortunately, it has been commonly observed that population structure is often quite low-dimensional, and only a few PCs frequently provide a good approximation of the covariance structure in human genetic variation data [30]. Therefore, we may hope that PCA could yield a reasonable approximation of the allele frequency space, and that *F*-statistics as measures of population structure may likewise be well approximated by the first few PCs.

### (c) Formal definition of principal component analysis

PCA is a common way of summarizing genetic data, and so a large number of variations of PCA exist, e.g. in how SNPs are standardized, how missing data are treated or whether we use individuals or populations as units of analysis [1,30]. The version of PCA I use here is set up such that the similarities to *F*-statistics are maximized, and does *not* reflect how PCA is most commonly applied to genome-scale human genetic variation datasets. In particular, I assume that a PCA is performed on unscaled, estimated population allele frequencies, whereas many applications of PCA are based on individual-level sample allele frequency, scaled by the estimated standard deviation of each SNP [30]. The differences this causes will be addressed in the discussion.

Let us again assume we have allele frequency data as above, but let us now assume we aggregate the allele frequency vectors *X*_*i*_ of many populations in a matrix **X** whose entry *x*_*il*_ reflects the allele frequency of the *i*th population at the *l*th genotype. If we have *S* SNPs and *n* populations, **X** will have dimension *n* × *S*. Since the allele frequencies are between zero and one, we can interpret each population *X*_*i*_ of **X** as a point in $R^{S}$.

PCA allows us to approximate the points in the high-dimensional allele frequency space by a *K*-dimensional subspace of the data. If all PCs are considered, *K* = *n* − 1, in which case the data are simply rotated. However, the historical processes that generated genetic variation often result in *low-rank* data [55], so that *K* ≪ *n* explains a substantial portion of the variation; for visualization, *K* = 2 is frequently used.

There are several algorithms that are used to perform PCAs, the most common one is based on singular value decomposition (e.g. [50]). In this approach, we first mean-centre **X**, obtaining a centred matrix **Y**

$$y_{il}=x_{il}-\mu_{l},$$

where *μ*_*l*_ is the mean allele frequency at the *l*th locus.

PCA can then be written as

2.6 $$Y=CX=(U\Sigma )V^{T}=PL,$$

where **C** = **I** − (1/*n*)**1** is a centring matrix that subtracts row means, with **I**, **1** the identity matrix and a matrix of ones, respectively. For any matrix **Y**, we can perform a singular value decomposition **Y** = **UΣV**^*T*^ which, in the context of PCA, is interpreted as follows: the matrix of principal components **P** = **UΣ** has size *n* × *n* and contains information about population structure. The SNP loadings **L** = **V**^*T*^ form an orthonormal basis of size *n* × *S*, its rows give the contribution of each SNP to each PC. It is often used to look for outliers, which might be indicative of selection (e.g. [56]). Alternatively, the PCs can also be obtained from an eigendecomposition of the covariance matrix **YY**^*T*^. This can be motivated from (2.6)

2.7 $$YY^{T}=PLL^{T}P^{T}=PP^{T},$$

since **LL**^*T*^ = **I**.

### (d) Connection between principal component analysis and *F*-statistics

#### (i) Principal components from *F*-statistics

PCA, as defined above, and *F*-statistics are closely related. It is a classical result that PCA is equivalent to multidimensional scaling using squared Euclidean distances [57]. Since *F*_2_-distances are squared Euclidean, we calculate the pairwise *F*_2_(*X*_*i*_, *X*_*j*_) between all *n* populations, and collect them in a matrix **F**_2_. Multidimensional scaling then proceeds by double-centring it, so that its row and column means are zero, and perform an eigen decomposition of the resulting matrix

2.8 $$PP^{T}=-\frac{1}{2}CF_{2}C.$$

Although we arrive at **P** from a very different angle, as long as we make the same choices about normalization and units of analysis, we will get the exact same results.

#### (ii) *F*-statistics in PCA space

By performing a PCA, we rotate our data to reveal the axes of highest variation. However, the dot product is invariant under rotation, and *F*-statistics can be thought of as dot products [49]. What this means is that we are free to calculate *F*_2_ either on the uncentred data **X**, the centred data **Y** or any other orthogonal basis such as the principal components **P**. Formally,

2.9 $$\begin{array}{rl} F_{2}(X_{i},X_{j}) & =\sum_{l=1}^{L}{(x_{il}-x_{\;jl})}^{2}\\ & =\sum_{l=1}^{L}{((x_{il}-\mu_{l})-(x_{\;jl}-\mu_{l}))}^{2}=F_{2}(Y_{i},Y_{j})\\ & =\sum_{k=1}^{n}{\left(p_{ik}-p_{\;jk}\right)}^{2}=F_{2}(P_{i},P_{j}).\; \end{array}$$

A derivation of this change-of-basis is given in appendix A, equation (A1). As *F*_3_ and *F*_4_ can be written as sums of *F*_2_ terms (equations (2.2*a*) and (2.2*b*)), analogous relations apply.

In most applications, we do not use all PCs, but instead truncate to the first *K* PCs, which explain most of the between-population genetic variation. Thus,

2.10 $$F_{2}(P_{i},P_{j})=\underset{{\hat{F_{2}}}^{\left(K\right)}(P_{i},P_{j})}{\underbrace{\sum_{k=1}^{K}{\left(p_{ik}-p_{\;jk}\right)}^{2}}}+\underset{\epsilon^{\left(K\right)}(P_{i},P_{j})}{\underbrace{\sum_{k=K+1}^{n}{\left(p_{ik}-p_{\;jk}\right)}^{2}}}.$$

In this notation, ${\hat{F_{2}}}^{\left(K\right)}$ is the approximation of *F*_2_ with only the first *K* PCs considered, and *ε*^(*K*)^ is the corresponding approximation error. I will omit the superscript of $\hat{F_{2}}$ when the exact number of PCs is not relevant. If we sum up the squared approximation errors over all pairs of populations in our sample, we obtain

2.11 $$\sum_{i,j}\epsilon^{\left(K\right)}{\left(P_{i},P_{j}\right)}^{2}=\sum_{i,j}{\left({\hat{F_{2}}}^{\left(K\right)}(P_{i},P_{j})-F_{2}^{\left(K\right)}(P_{i},P_{j})\right)}^{2}={\left\|F_{2}-\hat{F_{2}}\right\|}_{F}^{2},$$

where the Frobenius-norm ${\left\|\cdot \right\|}_{F}^{2}$ of a matrix is defined as the square root of the sum-of-squares of all its elements. This is precisely the function that is minimized in MDS [50]. In that sense, ${\hat{F}}_{2}^{\left(K\right)}$ is the optimal low-rank approximation of **F**_2_ for any *K* in that it minimizes the sum of approximation errors of all *F*_2_-statistics.

#### (iii) *F*-statistics and samples projected onto PCA

One of the easiest ways of dealing with missing data in PCA is to calculate the principal components (equation (2.6)) only on a subset of the data with no missingness, and then to *project* the lower quality samples with high missingness onto this PCA. The simplest way to do this is to note that

$$YL^{T}=PLL^{T}=P,$$

and so a new (centred) population *Y*_new_ can be projected onto an existing PCA simply by post-multiplying it with **L**^*T*^

$$P_{\mathrm{proj}}=Y_{\mathrm{new}}L^{T};$$

the *k*th entry of *P*_proj_ gives the coordinates of the new sample on the *k*th PC. However, it is likely that *Y*_new_ lies outside the variation of the original samples. In this case, there is a projection error

$${\left\|Y_{\mathrm{new}}-P_{\mathrm{proj}}L\right\|}^{2}=F_{2}(P_{\mathrm{proj}}L,Y_{\mathrm{new}}).$$

If we project with missing data, a similar projection can be used where we remove the rows from *Y*_new_ and **L** where data in *Y*_new_ is missing, and add a scaling factor [30].

Thus, if we compare the *F*-statistic of a projected sample, we have

2.12 $$\begin{array}{rl} F_{2}(X_{i},X_{\mathrm{new}}) & =F_{2}(Y_{i},Y_{\mathrm{new}})\\ & =F_{2}(P_{i},P_{\mathrm{proj}})+F_{2}(P_{\mathrm{proj}}L,Y_{\mathrm{new}})\\ & =\hat{F_{2}}(P_{i},P_{j})+\epsilon (P_{i},P_{j})+F_{2}(P_{\mathrm{proj}}L,Y_{\mathrm{new}}). \end{array}$$

The second row follows because the projection error and projection are orthogonal to each other. The main implication of equation (2.12) is that both truncation and projection introduce some error, and that $\hat{F_{2}}(P_{i},P_{j})$ will be a good approximation to *F*_2_(*P*_*i*_, *P*_*j*_) only if both errors are small.

## 3. Material and methods

The theory outlined in the previous section suggests that *F*-statistics have a geometric interpretation in PCA space, which can be approximated on PCA plots. In the next section, I explore this connection in detail, and illustrate it on two sample datasets that I briefly introduce here. Both are based on the analyses by Lazaridis *et al.* [58]. The data are from the Reich laboratory compendium dataset (v.44.3), downloaded from https://reich.hms.harvard.edu/allen-ancient-dna-resource-aadr-downloadable-genotypes-present-day-and-ancient-dna-data, using data on the ‘Human Origins’-SNP set (597,573 SNPs). SNPs with missing data in any population are excluded. The code used to write this paper, create all figures and analyses is available on doi:10.5281/zenodo.6424178.

### (a) ‘World’ dataset

This dataset is a subset of the ‘World Foci’ dataset of Lazaridis *et al.* [58], where I removed samples that are not permitted for free reuse. These populations span the globe and roughly represents global human genetic variation (638 individuals from 33 populations) As adjacent sampling locations are often thousands of kilometres apart, I speculate that gene flow between these populations may not be particularly common; and their structure may therefore be well approximated by an admixture graph. A file with all individuals used and their assigned population is given in electronic supplementary material, File S1.

### (b) Western Eurasian dataset

This dataset of 1119 individuals from 62 populations contains present-day individuals from the Eastern Mediterranean, Caucasus and Europe. Lazaridis *et al.* [58] used this dataset as a basis of comparison for ancient genetic analyses of Western Eurasian individuals, and PCAs based on similar sets of samples have been used in many other ancient DNA studies (e.g. [59,60]). Genetic differentiation in this region is low and closely mirrors geography [45]. I thus speculate that gene flow between these populations is common [61], and a discrete model such as a tree or an admixture graph might be a rather poor reflection of this data. A file with all individuals used and their assigned population is given in electronic supplementary material, File S2.

### (c) Computing *F*-statistics and PCA

All computations are performed in R. I use admixtools 2.0.0 (https://github.com/uqrmaie1/admixtools) to compute *F*-statistics. To obtain a PC-decomposition, I first calculate all pairwise *F*_2_-statistics, and then use equation (2.8) and the eigen function to obtain the PCs. The right-hand-side matrix of equation (2.8) is expected to have non-negative eigenvalues (i.e. −**CF**_2_**C** is positive-semidefinite). However, when *F*_2_-statistics are estimated from data, sampling noise might make some of them slightly negative, which would lead to imaginary PCs. I avoid this by setting all negative eigenvalues to zero.

## 4. Results

The transformation from the previous section allows us to consider the geometry of *F*-statistics in PCA space. The relationships we will discuss formally only hold if we use all PCs. However, the appeal of PCA is that frequently, only a very small number *K* ≪ *n* of PCs contain most information that is relevant for population structure, in which case the geometric interpretations become very simple. Thus, throughout the schematic figures, I assume that two PCs are sufficient to characterize population structure. In the data applications, I evaluate how deviations of this assumption may manifest themselves in PCA plots.

### (a) *F*_2_ in PC space

The *F*_2_-statistic is an estimate of the squared allele-frequency distance between two populations. On a tree (figure 1*a*), this corresponds to the branch between two populations. In allele-frequency space, it corresponds to the squared Euclidean distance, and thus reflects the intuition that closely related populations will fall close to each other on a PCA plot, and have low pairwise *F*_2_-statistics. However, since *F*_2_ can be written as a sum of squared (non-negative) terms for each PC (equation 2.9), the distance on a PCA plot will always be an underestimate of the full *F*_2_ distance. Thus, PCA might project two populations with high *F*_2_ distance very close to each other, which would indicate that these particular PCs are not suitable to understand and visualize the relationship between these particular populations, and likely more PCs need to be investigated to understand how these populations are related to each other. In converse, populations that are distant on the first few PCs are guaranteed to also have a large *F*_2_-distance, since the distance contributed from the omitted PCs cannot be negative.

### (b) When are admixture-*F*_3_-statistics negative?

Consider again the admixture scenario in figure 2*a*, where population *X*_*y*_ is the result of a mixture of *X*_2_ and *X*_3_, and subsequent drift changes the allele frequencies of the admixed population from *X*_*y*_ to *X*_*x*_. How is such a scenario displayed on a PCA? Since the allele frequencies of *X*_*y*_ are a linear combination of *X*_2_ and *X*_3_, it will lie on the line segment connecting these two populations (figure 2*b*), at a location predicted by the admixture proportions. Subsequent drift will change the allele frequencies of *X*_*y*_ (to say, *X*_*x*_), and so in general it might fall on a different point on a PCA plot. An exception occurs when *X*_*x*_ (and no other populations related to *X*_*x*_) are not part of the construction of the PCA, so that *X*_*x*_ − *X*_*y*_ is orthogonal to all PCs, i.e.

$$\langle X_{x}-X_{y},X_{i}-X_{j}\rangle =\langle X_{x}-X_{y},P_{i}\rangle =0$$

for all populations *i*, *j* ≤ *n*. In this case, *X*_*x*_ and *X*_*y*_ project to the same point, and the location on the PCA can directly be used to predict the admixture proportions [47,49,62]. However, if either *X*_*x*_ is included in the construction of the PCA, or if some gene flow occurred between *X*_*x*_ and any of the populations used to construct the PCA, *X*_*x*_ and *X*_*y*_ may project on different spots (figure 2*b*).

Thus, a natural question to ask is given two source populations *X*_2_, *X*_3_, can we use PCA to predict which populations might have negative *F*_3_-statistics? This condition can be written as

4.1 $$\begin{array}{rl} 2F_{3}(X_{x};X_{2},X_{3}) & =2\langle X_{x}-X_{2},X_{x}-X_{3}\rangle \\ & ={\left\|X_{x}-X_{2}\right\|}^{2}+{\left\|X_{x}-X_{3}\right\|}^{2}-{\left\|X_{2}-X_{3}\right\|}^{2}<0. \end{array}$$

By the Pythagorean theorem, *F*_3_ = 0 if and only if *X*_2_, *X*_3_ and *X*_*x*_ form a right-angled triangle. The associated region where *F*_3_ = 0 is an *n*-sphere (or a circle in two dimensions) with diameter $\bar{X_{2}X_{3}}$ (the overline denotes a line segment). *F*_3_ is negative when the triangle is obtuse, i.e. *X*_*x*_ could be considered admixed if it lies inside the *n*-ball with diameter $\bar{X_{2}X_{3}}$ (figure 1*b*, equation (A2)).

### (c) *F*_3_ and dimensionality reduction

**Figure 3.**
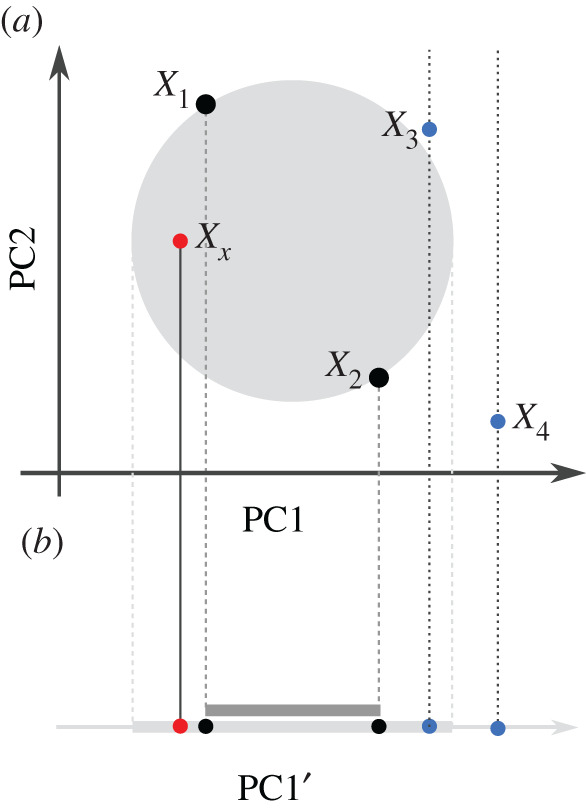
Admixture-*F*_3_-statistic and dimensionality reduction. (*a*) The grey circle is defined by *X*_1_ and *X*_2_ and reflects the area for which *F*_3_ is negative, i.e. *F*_3_(*X*_*x*_; *X*_1_, *X*_2_) < 0 < *F*_3_(*X*_3_; *X*_1_, *X*_2_) ≤ *F*_3_(*X*_4_; *X*_1_, *X*_2_). (*b*) Projection on one dimension. The full rejection region (light grey bar) is given by the boundaries of the circle in *a*, and is larger than that predicted from the distance between *X*_1_ and *X*_2_ (dark grey bar) on PC1 alone. Points like *X*_*x*_ with negative *F*_3_ are guaranteed to lie in the light grey (but not dark grey) region. *X*_3_ also projects onto the grey bar, even though it is outside the circle and *F*_3_ is positive, and it would also lie inside the positive area if projected onto PC2. However, points such as *X*_4_ that project outside the rejection region have *F*_3_ > 0, since the associated projection line does not intercept the circle. (Online version in colour.)

If we project this *n*-ball on a two-dimensional plot, $\bar{X_{2}X_{3}}$ will usually not align with the PCs; thus the ball may be somewhat larger than it appears on the plot. This geometry is perhaps easiest visualized on the example of projecting *F*_3_(*X*_*x*_; *X*_1_, *X*_2_) from a two-dimensional space onto a single dimension (figure 3). In that example, the distance between *X*_1_ and *X*_2_ has a substantial contribution from PC2, and so the negative region (light grey) is larger than what would be predicted from just one dimension (dark grey bar), but if ${\hat{F}}_{3}\approx F_{3}$, the two areas would be very close. Thus, if considering a reduced-dimension PCA plot, some points (such as *X*_3_) may project inside the negative region, but have positive *F*_3_ because they are outside the *n*-ball in higher dimensions. The converse interpretation is more strict: if a population lies outside the circle on *any* projection, *F*_3_ is guaranteed to be bigger than 0 (see equation (A4) in the appendix). An intuitive example is given by *X*_4_ in figure 3: all points projecting to the same point on figure 3*b* as *X*_4_ lie outside the circle.

#### (i) Example

**Figure 4.**
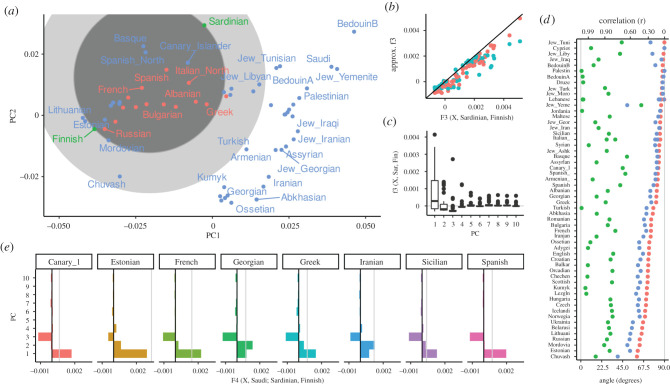
PCA and *F*-statistics for the Western Eurasian dataset. (*a*) PCA biplot; the light grey circle denotes the region for which *F*_3_(*X*; Sardinian, Finnish) may be negative, the dark circle is based on just the first two PCs. Populations for which *F*_3_ is negative are coloured in red. (*b*) *F*_3_ approximated with two (blue) and 10 (red) PCs versus the full spectrum. (*c*) Boxplot of contributions of PCs 1–10 to each *F*_3_-statistic. (*d*) Projection angle and correlation interpretation of *F*_4_(*X*, Saudi; Sardinian, Finnish) based on two PCs (green), three PCs (blue) or full data (red). (*e*) Contribution of the first 10 PCs to select *F*_4_-statistics, with the first three PCs containing the majority of contributions. (Online version in colour.)

As an example, I visualize the admixture statistic *F*_3_(*X*; Sardinian, Finnish), on the first two PCs of the Western Eurasian dataset (figure 4*a*). In this case, the projected *n*-ball (light grey) and circle based on two dimensions (dark grey) have similar sizes. However, several populations that appear inside the circles (e.g. Basque, Canary Islanders) have, in fact, positive *F*_3_ values, so they lie outside the *n*-ball. This reveals that the first two PCs do not capture all the genetic variation relevant for European population structure. Consequently, approximating *F*_3_ by the first two or even 10 PCs (figure 4*b*) only gives a coarse approximation of *F*_3_, and from figure 4*c* we see that many higher PCs contribute to *F*_3_ statistics.

However, many populations, particularly from Western Asia and the Caucasus, on the right-hand side of the plot, fall outside the circle. This allows us to immediately conclude that their *F*_3_-statistics must be positive, and we should not consider them as a mixture between Sardinians and Fins.

### (d) Outgroup-*F*_3_-statistics as projections

**Figure 5.**
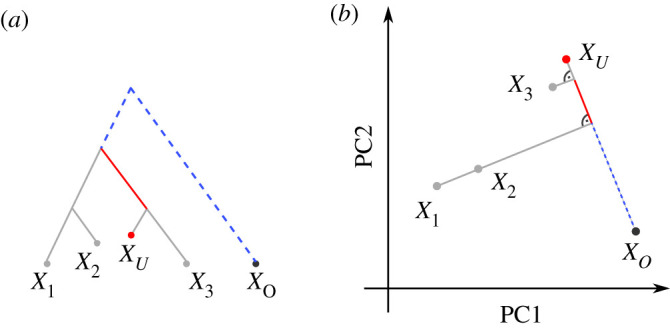
Outgroup-*F*_3_-statistics. Interpretation of outgroup-*F*_3_-statistic on a tree (*a*) and PCA plot (*b*). The highlighted segment represents *F*_3_(*X*_*O*_; *X*_*U*_, *X*_3_) and the dashed segment reflects *F*_3_(*X*_*O*_; *X*_*U*_, *X*_1_) and *F*_3_(*X*_*O*_; *X*_*U*_, *X*_2_), which have the same value. (Online version in colour.)

A common application of *F*_3_-statistics is, given an unknown sample *X*_*U*_, to find the most closely related population among a reference panel (*X*_*i*_) [63]. This is done using an *outgroup*-*F*_3_-statistic *F*_3_(*X*_*O*_; *X*_*U*_, *X*_*i*_), where *X*_*O*_ is an outgroup. The reason an outgroup is introduced is to account for differences in sample times and additional drift in the reference populations (figure 5*a*). The outgroup-*F*_3_-statistic *F*_3_(*X*_*O*_; *X*_*U*_, *X*_3_) represents the branch length from *X*_*O*_ to the common node between the three samples in the statistic, and the closer this node is to *X*_*U*_, the longer the branch and hence the larger the *F*_3_-statistic.

To make sense of outgroup-*F*_3_-statistics in the PCA context, I use the association of *F*_3_-statistics to projections (equation 2.5): on a PCA plot, we can visualize this *F*_3_-statistic as the projection of the vector *X*_*i*_ − *X*_*O*_ onto *X*_*U*_ − *X*_*O*_

$${\;proj}_{X_{U}-X_{O}}(X_{i}-X_{O})=F_{3}(X_{O};X_{U},X_{i})\frac{X_{U}-X_{O}}{F_{2}(X_{O};X_{U})}.$$

Of the right-hand-side terms, only the *F*_3_ term depends on the *X*_*i*_. The fraction can be thought of as a normalizing constant, so the *F*_3_-statistic is proportional to the length of the projected vector. This means that the outgroup-*F*_3_-statistic is largest for whichever *X*_*i*_ projects furthest along the axis from the outgroup to the unknown population; in figure 5, this is *X*_3_.

#### (i) Example

**Figure 6.**
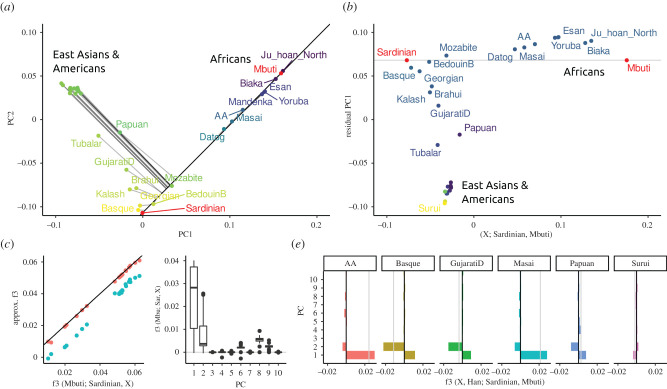
PCA and *F*-statistics for the World dataset. (*a*) Visualization of outgroup-*F*_3_-statistic *F*_3_(Mbuti; Sardinian, *X*) on a PCA biplot. The colour of points correspond to the value of the *F*_3_-statistic, with brighter yellows indicating higher values, i.e. higher similarity to Sardinians. The *F*_3_-projection axis is given by a black line, the projection of populations onto this axis by thin grey lines. In the full allele frequency space, these projection are orthogonal to the axis. (*b*) Projection along the axis Sardinian-Mbuti (*X*-axis), and PCA on residual of this projection (PC1 on *Y*-axis, PC2 as colouring). (*c*) Approximation of *F*_3_(Mbuti; Sardinian, *X*) using the first two (blue) and first 10 (red) PCs, respectively. (*d*) Contributions of first 10 PCs to all statistics of the form *F*_3_(Mbuti; Sardinian, *X*). (*e*) Contributions of the first ten PCs to select *F*_4_-statistics. (Online version in colour.)

In figure 6*a*, I use the World dataset to visualize the outgroup-*F*_3_-statistic *F*_3_ (Mbuti; Sardinian, *X*_*i*_), in i.e. a statistic that aims to find the population most closely related to Sardinian (a Mediterranean island), assuming the Mbuti are an outgroup to all populations in the dataset. On a PCA, we can interpret this *F*_3_-statistic as the projection of the line segment from Mbuti to population *X*_*i*_ onto the line through Mbuti and Sardinians (black line). For each population, the projection is indicated with a grey line. In the full data space, this line is always orthogonal to the segment Mbuti-Sardinian, but on the plot (i.e. the subspace spanned by the first two PCs), this is not necessarily the case. The colouring is based on the *F*_3_-statistic calculated from all the data, with brighter values indicating higher *F*_3_-statistics. In this case, the first two PCs approximate the *F*_3_-statistic very well: particularly, the samples from East Asia and the Americas project almost orthogonally, suggesting that most of the genetic variation relevant for this analysis is captured by these first two PCs. We can quantify this and find that the first two PCs slightly underestimate the absolute value of *F*_3_ (figure 4*c*), but keep the relative ordering. I also find that many PCs, e.g. PCs 3–5, 7 and 10, have almost zero contribution to all *F*_3_-statistics (figure 4*d*), PCs 6, 8 and 9 having a similar non-zero contribution for almost all statistics, likely because these PCs explain within-African variation.

### (e) *F*_4_-statistics as angles

One interpretation of *F*_4_ on PCA plots is similar to that of *F*_3_: as a projection of one vector onto another, with the difference that now all four points may be distinct. *F*_4_-statistics that correspond to an internal branch in a tree (as in figure 1*c*) can be interpreted as being proportional to the length of a projected segment on a PCA plot, again with the caveat that we need to scale it by a constant. If the *F*_4_-statistic corresponds to a branch that does not exist in the tree (figure 1*d*), then, from the tree interpretation, we expect *F*_4_(*X*_1_, *X*_2_; *X*_3_, *X*_4_) = 0 implying that the vectors *X*_1_ − *X*_2_ and *X*_3_ − *X*_4_ are orthogonal to each other, i.e. that *X*_1_ and *X*_2_ map to the same point on the projection axis $\bar{X_{3}X_{4}}$. In the case of an admixture graph, this is no longer the case: Both population *X*_*y*_ and *X*_*x*_ in figure 2*d* do *not* map to the same point as *X*_1_ or *X*_2_ do, implying that statistics of the form *F*_4_(*X*_1_, *X*_*x*_; *X*_3_, *X*_4_) ≠ 0.

Since *F*_4_ is a covariance, its magnitude lacks an interpretation. Therefore, commonly, correlation coefficients are used, as there, zero means independence and one means maximum correlation. For *F*_4_, we can write

4.2 $$\text{Cor}(X_{1}-X_{2},X_{3}-X_{4})=\frac{F_{4}(X_{1},X_{2};X_{3},X_{4})}{\left\|X_{1}-X_{2}\right\|{\left\|X_{3}-X_{4}\right\|}^{2}}=\cos(\phi ),$$

where *ϕ* is the angle between *X*_1_ − *X*_2_ and *X*_3_ − *X*_4_. Thus, independent drift events lead to cos (*ϕ*) = 0, so that the angle is 90${\;}^{\circ }$, whereas an angle close to zero (cos (*ϕ*) ≈ 1) means most of the genetic drift on this branch is shared.

#### (i) Example

To illustrate the angle interpretation, I return to the Western Eurasian data. The PCA biplot shows two roughly parallel clines (figure 4*a*), a European gradient (from Sardinian to Finnish and Chuvash), and an Asian cline from Arab populations (top right) to the Caucasus (bottom right). This is quantified in figure 4*d*, where I plot the angle corresponding to *F*_4_(*X*, Saudi; Sardinian, Finnish). For most Asian populations, using two PCs (green points) gives an angle close to zero, corresponding to a correlation coefficient between the two clines of *r* > 0.9. Just adding a third PC (blue), however, shows that the clines are not, in fact, parallel, and the correlation for most populations is low. The finding that three PCs are necessary to explain this data can also be seen from the spectrum of these *F*_4_-statistics (figure 4*e*), which have high contributions from the first three PCs. Both results indicate that adding a third PC would give a much better description of the data, and the relationship between within-European variation to Saudis in particular.

### (f) Other projections

So far, I used equation (2.9) to interpret *F*-statistics on a PCA plot, but the argument holds for *any* orthonormal projection in the data space. This is useful in particular for estimates of admixture proportions, which are often done as projections into a low-dimensional reference space defined by *F*-statistics [38,40,49,53].

For example, a common way to estimate admixture proportion *α* of *X*_1_ is the *F*_4_-ratio

4.3 $$\alpha =\frac{F_{4}(R_{1},R_{2};X_{X},X_{1})}{F_{4}(R_{1},R_{2};X_{2},X_{1})}=\frac{{\;proj}_{\left[R_{1}-R_{2}\right]}X_{X}-X_{1}}{{\;proj}_{\left[R_{1}-R_{2}\right]}X_{2}-X_{1}},$$

which can be interpreted as projecting *X*_*X*_ − *X*_1_ and *X*_2_ − *X*_1_ onto *R*_1_ − *R*_2_, and the ratio of the lengths gives the proportion of *X*_*x*_ contributed by *X*_1_ [49].

**Figure 7.**
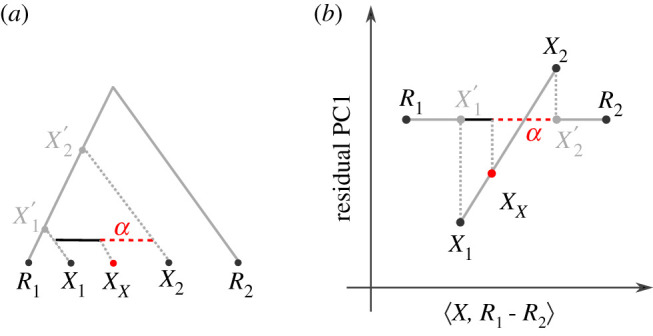
Admixture proportion estimates. (*a*) Visualization of the admixture graph scenario used to estimate the proportion *α* contributed from *X*_1_ to *X*_*X*_, using references *R*_1_ and *R*_2_. The full grey line corresponds to the projection axis, and the dotted grey lines correspond to the branches ignored in the projections. The admixture proportion *α* corresponds to the length of the dashed red line relative to the black line between *X*_1_ and *X*_2_. (*b*) The same scenario, but in Euclidean space, *X*_1_, *X*_2_ and *X*_*X*_ align on a line both in the (low-dimensional approximation of the) residual space and on the projection axis. (Online version in colour.)

The admixture graph motivating this statistic is visualized in figure 7*a*, and the PCA-like interpretation in figure 7*b*. In both panels, the solid grey line is the projection axis, and the dotted line gives the residual, i.e. the branches or genetic variation that is ignored by the projection.

The PCA-like projection can be used to visualize admixture proportions, as the horizontal position of *X*_*X*_ relative to *X*_1_ and *X*_2_ (red dashed line versus black line) directly represents the estimated admixture proportion *α*. In addition, the residuals can be used to verify assumptions of the admixture graph model. In particular, since *X*_*X*_ arises as a linear combination of *X*_1_ and *X*_2_, if admixture is recent we might expect the three populations to be collinear; if they are not this means that either of the populations experienced gene flow from some other population which might bias results [53].

In addition, the external tree branches *X*_1_ − *X*_1_′ and *X*_2_ − *X*_2_′ are disjoint which means they should be orthogonal. On a one-dimensional residual plot (figure 7*b*), this cannot be verified, but the statistic

4.4 $$F_{4}(X_{1},X_{1}^{\prime };X_{2},X_{2}^{\prime })=0$$

can be calculated for all samples.

#### (i) Example

I use the World dataset as an example, using Sardinian and Mbuti as references populations (figure 6*b*). The data are the same as in the PCA (figure 6*a*), but it is now rotated such that the axis between the reference population (black line in figure 6*a*) is aligned with the *x*-axis. For any pair of populations *X*_1_ *X*_2_, their horizontal projection distance reflects *F*_4_ (Sardinian, Mbuti; *X*_1_, *X*_2_) and the relative horizontal distance corresponds exactly to *F*_4_-ratio admixture estimates. For many sets of populations, this is of course not sensible, and just looking at the first PC of the residual shows many examples where the populations are not collinear. For example, on the *x*-axis, the South American Surui are between Papuans and Georgians, but since the Surui clearly are not on the line between Papuans and Georgians, this cannot be the result of admixture.

## 5. Discussion

Particularly for the analysis of human genetic variation with a large number of individuals with heterogeneous relationships, *F*-statistics are a powerful tool to describe population genetic diversity. Here, I show that the geometry of *F*-statistics [49] leads to a number of simple interpretations of *F*-statistics on a PCA plot.

### (a) The geometry of admixture

Previous interpretation of PCA in the context of population genetic models have focused on explicit models and aimed at directly interpreting the PCs in terms of population genetic parameters [16,23,46,48]. My interpretation here is different in that the utility of PCA is to simplify the geometry of the data, rather than attributing meaning to the produced PCs. One consequence is that the results here are less directly impacted by sample ascertainment, sample sizes or number of PCs, which are common concerns in the interpretation of PCA [23,46–48]; adding more PCs will provide a successively better approximation of the *F*-statistics.

The two datasets I analysed here suggest that two PCs (for the World dataset), and three PCs (for the Western Eurasian data), respectively, already provide a very good approximation for *F*_4_-statistics (figures 4*e* and 6*e*), reflecting the observation that frequently the first few PCs provide a good approximation of the overall population structure. On the other hand, for admixture *F*_3_-statistics, more PCs are needed (figure 4*b*). This is likely due to PCA approximating the global structure in the dataset; statistics that only involve distantly related populations will only require a few PCs for good approximations, whereas statistics that contain a term measuring local variation, such as *F*_3_-statistics or *F*_4_ between closely related populations will require more PCs for good approximations, because local variation is often found on higher PCs.

My focus on the geometry of the data allows for direct and quantitative comparisons between *F*-statistic-based results and PCA biplots. As PCA is often ran in an early step in data analysis, this may aid in generation of hypotheses that can be more directly evaluated using generative models, typically using a lower number of populations. It also allows reconciling apparent contradictions between *F*-statistics and PCA plots. In many cases, differences between the two data summaries will be due to variation on higher PCs. In this case, plotting additional PCs, or further subsetting the data to a more local set of populations seems prudent.

### (b) Assumptions

In addition to the selection of PCs, the other cause for disagreements between *F*-statistics and PCA are differences in assumptions. The version of PCA I use for my analyses is chosen such that the similarities to *F*-statistics are maximized. In particular, I assume here that (i) we have no missing data, (ii) SNPs are equally weighted, (iii) that individuals can be grouped into populations and (iv) we use estimated allele frequencies. By contrast, most data analyses have to grapple with missing data, SNPs are often weighted according to their allele frequencies, and observed, individual-level genotypes are used as the basis of PCA.

#### (i) Missing data

The matrix decompositions underlying PCA assume complete data, and thus cannot be used when some data is missing [50]. As missing data are a very common practical problem, there is a large number of algorithms for imputing missing data. The simplest approach is to replace missing data with zeros (as implemented e.g. in [30]), but more sophisticated algorithms exist to ‘learn’ the missing values from surrounding data (e.g. [64,65]). By contrast, missing data in *F*-statistics is most commonly handled by estimating a standard error by resampling along the genome [40], and so missing data results in larger standard errors.

These strategies are distinct, and reflect the original purposes of the approaches. For statistical tests based on *F*-statistics, we wish to isolate a set of three or four populations and get our best guess based on just that subset of data. By contrast, methods for PCA can leverage the additional individuals, and thus will likely result in more accurate estimates.

However, the way we handle missing data is not tied into the method. For example, we could evaluate the robustness of a PCA by resampling data. Similarly, the theory developed here suggests that we could obtain accurate *F*-statistics with missing data by first performing a PCA using a method that handles missing data, and then calculate *F*-statistics from these PCs.

#### (ii) Normalization

In PCA, SNPs are typically normalized to have expected variance of one, a step that is omitted in calculating *F*-statistics [40]. The *F*-statistic framework assumes that each SNP is an identically distributed (but not independent) random variable, which holds regardless of weighting. Thus, normalization of SNPs is largely a matter of convention; for *F*-statistics the dependency on additional samples (through mean allele frequencies) is often unwanted, but could be advantageous for tools that aim to do joint inference from many *F*-statistics such as qpAdm [38,40]. As genetic differentiation between human populations is low, the normalization used may matter little in practice, but could be explored in future work [28].

#### (iii) Estimated versus observed allele frequencies

The third difference between *F*-statistics and PCA is on the usage of estimated allele frequencies versus individual-based genotypes. The fact that PCA does not distinguish between sampling error and the underlying structure is a well-known drawback of PCA, and applying the theory presented here to individual-based PCA would result in *F*-statistics that incorporate some sampling noise. Probabilistic PCA is one class of approaches that aim to separate the population structure from sampling noise (e.g. [66]). It seems likely that probabilistic PCA would yield a representation of the data that is more closely aligned with *F*-statistics than regular PCA.

#### (iv) Individual versus population-based analyses

The final issue is that PCA is commonly run on individual-based data, whereas *F*-statistics often group individuals into populations. However, population-based PCA has been the default in the past [1], and *F*-statistics are often applied to individuals (e.g. [25,67,68]). Often, an individual-based PCA is used to justify grouping individuals into populations; i.e. individuals that form a tight cluster on a PCA plot have similar relationships to everyone else in the dataset, and can thus be treated as a unit of analysis. Thus, if the assumptions are satisfied, *F*-statistics for individual-based and population-based analyses are expected to be very similar. PCA, on the other hand, is strongly impacted by the number of individuals from each population (e.g. [47]); as each individual is weighted equally, variation related to populations with many samples will be overrepresented on the first PCs.

#### (v) Summary

Motivated by *F*-statistics, the PCAs I consider here are based on estimated population allele frequencies, whereas most genome-scale studies of human genetic variation use observed individual-based allele frequencies that are normalized by overall allele frequencies. Thus, some care will be required to directly extend the interpretations developed here to individual-based PCAs. However, the differences are largely due to conventions, and particularly for studies where the description of population structure is a major focus, results might be easier to interpret if conventions regarding missing data, normalization and estimation of allele frequencies are used consistently between *F*-statistics and PCA.

### (c) The apportionment of human diversity

Most genetic variation in humans is shared between all of us, but the around 15% that can be explained by population structure can be leveraged to study our history and diversity in great detail [1,69,70]. For some datasets, it is possible to predict an individuals’ origin at a resolution of a few hundred kilometres [45,71], and direct-to-consumer-genetics companies are using this variation to analyse the genetic data of millions of customers.

However, understanding, conceptualizing and modelling this variation is far from trivial, particularly in a historical context in which mistaken ideas about human variation have been used to justify racist, eugenic and genocidal policies. Lewontin’s landmark 1972 paper on the apportionment of human genetic diversity was one of the first to quantify how little of between-population genetic variation could be attributed to ‘racial’ continental-scale groupings [70]. Over the last five decades, this view has been corroborated, refined and extended many times [1,72–74].

From a practical perspective, formulating hypotheses and designing studies in terms of discrete populations with ‘uniform’ genetic backgrounds is often sensible, as it enables e.g. prediction of phenotypes [75,76], inference of demographic parameters, and schematic models of human genetic history [40]. In a similar vein, when interpreting *F*-statistics in the context of admixture graphs, we make the implicit assumption that populations are discrete, related as a graph, and that gene flow between populations is rare [38,40]. However, these simplifications do come at a cost, both in terms of model violations that may invalidate statistical results, and in terms of deemphasizing that people do not rigidly fall into predefined genetic groups.

In many parts of the world, and particularly at more local scales, distinctions between populations begin to blur, and everyone could be considered admixed to some degree [77]. This provides a challenge for interpretation, as most *F*_3_ and *F*_4_-statistics will indicate departures from treeness. A naive interpretation of the *F*-statistics from my Eurasian example (figure 4*a*) would identify a substantial fraction of Europeans as (significantly) admixed between Finnish and Sardinians. By contrast, PCA reveals that the variation in this dataset is not due to a single event, and so an arguably better description of the dataset is one where Finnish and Sardinians lie on opposite ends of a more gradually structured population.

Thus, a more general way we could think about modelling population structure using *F*-statistics is as identifying orthogonal drift components. In a tree model, orthogonality arises because changes in allele frequencies on distinct branches of the tree are independent from each other (and high-dimensional random vectors are almost surely orthogonal). The classical model of admixture as a result of contact between long-separated and isolated populations is one of potentially many demographic models that results in non-orthogonality; gene flow of any kind will result in correlated genetic drift, and hence in non-zero *F*_3_ or *F*_4_-statistics.

Thinking of population structure in terms of orthogonal components may be abstract, but it is quite similar to how PCA is sometimes interpreted. In a PCA, we frequently make the informal observation that a particular PC is associated with variation within a specific region, or separates out distinct populations [1]. These associations are not exact, and are impacted e.g. by the size and composition of the analysed dataset. On the other hand, we could use *F*-statistics to formally test orthogonality, or to quantify correlations. Motivated by PCA, we could set up *F*_4_ as tests of orthogonality due to either space (distinct populations evolve independently) or scale (i.e. between-population diversity is independent of within-population diversity). This slight generalization of *F*-statistics could allow us to reframe many questions about gene flow or divergence that are currently asked as tests of orthogonality, without assuming that lineages or admixture events are discrete.

## 6. Conclusion

*F*-statistics and PCA have both proven to be tremendously useful to study, visualize and test aspects of population structure. Here, I show that these approaches are closely connected, and highlight a few implications. First, PCA and *F*-statistics should never be treated as independent analyses. If they agree, this can be used as a sanity check that no major assumptions about, e.g. population groupings, are violated, but the underlying biological relationships investigated are the same in both approaches.

If sufficiently many PCs are considered, both *F*_3_ and *F*_4_-statistics do have simple interpretations in PCA space. If used as a test for admixture, *F*_3_ corresponds to testing whether the admixed population lies in an *n*-sphere between the potential source population in PCA space. This result makes the informal notion that an admixed population should lie between its sources on a PCA plot more precise. Furthermore, I show that while it is necessary for an admixed population to lie between its sources on a PCA plot, this is not a sufficient condition and unadmixed populations may also project inside the *n*-sphere. By contrast, if a population falls outside the *n*-sphere on any PCA plot, this is sufficient to determine that this population has a positive *F*_3_ statistic.

Interpretations of the outgroup-*F*_3_-statistics and *F*_4_-statistics in a PCA framework rely heavily on the geometric concepts of projections and orthogonality: in a tree or admixture graph framework, we can loosely interpret *F*_4_(*A*, *B*; *C*, *D*) as the overlap of the path from *A* to *B* onto the path from *C* to *D*, or equivalently as the projection of *A* − *B* onto *C* − *D*; only edges shared between the two paths will contribute non-zero amounts to this statistic. This interpretation holds when we replace the populations by points in PCA space, and enables us to study population models beyond discrete graphs. Thus, the geometric framework enables us to expand the application of *F*-statistics beyond current uses, allows us to better understand the meaning of these statistics and helps us to avoid overinterpretations, particularly in cases when population structure is continuous.

## Data Availability

All results are based on public data. The code used to obtain all results is available at doi:10.5281/zenodo.6424178. The data are provided in electronic supplementary material [78].

## Appendix A. Derivations

Depending on a reader's background in linear algebra, these results may appear elementary; I include them here for reference and because they were not obvious to me at the onset of this project.

### (a) *F*-statistics are invariant under a change-of-basis

A 1 $$\begin{array}{rl} F_{2}(X_{i},X_{j}) & =\sum_{l=1}^{S}{((x_{il}-\mu_{l})-(x_{\;jl}-\mu_{l}))}^{2}=F_{2}(Y_{i},Y_{j})\\ & =\sum_{l=1}^{S}{(\sum_{k}L_{kl}P_{ik}-\sum_{k}L_{kl}P_{\;jk})}^{2}\\ & =\sum_{l=1}^{S}{\left(\sum_{k}L_{kl}(P_{ik}-P_{\;jk})\right)}^{2}\\ & =\sum_{l=1}^{S}\left(\sum_{k}L_{kl}^{2}{\left(P_{ik}-P_{\;jk}\right)}^{2}+2\sum_{k\neq k^{\prime }}L_{kl}L_{k^{\prime }l}{\left(P_{ik}-P_{\;jk^{\prime }}\right)}^{2}\right)\\ & =\sum_{k}\underset{1}{\underbrace{\left(\sum_{l=1}^{S}L_{kl}^{2}\right)}}{\left(P_{ik}-P_{\;jk}\right)}^{2}\\ & \;+2\sum_{k\neq k^{\prime }}\underset{0}{\underbrace{\left(\sum_{l=1}^{S}L_{kl}L_{k^{\prime }l}\right)}}{\left(P_{ik}-P_{\;jk^{\prime }}\right)}^{2}\\ & =\sum_{k}{\left(P_{ik}-P_{\;jk}\right)}^{2}. \end{array}$$

In summary, the first row shows that *F*_2_ on the centred data will give the same results (as distances are invariant to translations); in the second row, we apply the PC decomposition. The third row is obtained from factoring out *L*_*lk*_. Row four is obtained by multiplying out the sum inside the square term for a particular *l*. We have *k* terms when for $\left(\begin{array}{c} k\\ 2 \end{array}\right)$ terms for different *k*s. Row five is obtained by expanding the outer sum and grouping terms by *k*. The final line is obtained by recognizing that **L** is an orthonormal basis, where dot products of different vectors have lengths zero.

Note that if we estimate *F*_2_, unbiased estimators are obtained by subtracting the population heterozygosities *H*_*i*_, *H*_*j*_ from the statistic. As these are scalars, they do not change above calculation.

### (b) The region of negative *F*_3_-statistics is an *n*-ball

Without loss of generality, assume that *X*_1_ = (*r*, 0, 0, …) and *X*_2_ = (−*r*, 0, 0, …), and let us assume that *X*_*x*_ has coordinates (*x*_1_, *x*_2_, …, *x*_*S*_). Assuming *F*_3_(*X*_*x*_; *X*_1_, *X*_2_) = 0, equation (4.1) becomes

A 2 $$\begin{array}{rl} 2F_{3}(X_{x};X_{1},X_{2}) & ={\left\|X_{x}-X_{1}\right\|}^{2}+{\left\|X_{x}-X_{2}\right\|}^{2}-{\left\|X_{1}-X_{2}\right\|}^{2}=0\\ & =\left[{\left(x_{1}-r\right)}^{2}+\sum_{i=2}^{S}x_{i}^{2}\right]+\left[{\left(x_{1}+r\right)}^{2}+\sum_{i=2}^{S}x_{i}^{2}\right]-4r^{2}\\ & =2\left[\sum_{i=1}^{S}x_{i}^{2}+r^{2}+x_{1}r-x_{1}r\right]-4r^{2}\\ \;\;F_{3}(X_{x};X_{1},X_{2}) & =-r^{2}+\sum_{i=1}^{S}x_{i}^{2}=-r^{2}+{\left\|X_{x}\right\|}^{2}=0, \end{array}$$

which is the equation of an *n*-sphere with radius *r* and centre at the origin, as assumed from the placing of *X*_1_ and *X*_2_. Now, assume that *F*_3_ is negative, i.e. *F*_3_(*X*_*x*_; *X*_1_, *X*_2_) = −*k* < 0. Moving *r*^2^ to the left, we obtain

A 3 $$r^{2}-k={\left\|X_{x}\right\|}^{2},$$

which is another *n*-sphere with a smaller radius, showing that all points inside the *n*-sphere will have negative *F*_3_ values.

### (c) If a population lies outside the circle of this *n*-sphere in any two-dimensional projection, *F*_3_ is positive

Assume the centre of the *n*-sphere *C* = (*X*_1_ + *X*_2_/2) = (*c*_1_, *c*_2_, …, *c*_*S*_), and *X*_*x*_ = (*x*_1_, *x*_2_, …, *x*_*S*_). Then,

A 4 F3(Xx;X1,X2)=‖Xx−C‖2−r2 =(x1−c1)2+(x2−c2)2⏟>r2+∑i=3S(xi−ci)2⏟≥0−r2  >0.

The condition (*x*_1_ − *c*_1_)^2^ + (*x*_2_ − *c*_2_)^2^ > *r*^2^ is satisfied whenever *X*_*x*_ is outside the circle obtained from projecting the *n*-sphere on the first two dimensions. An analogous argument applies for any low-dimensional representation.
